# Use and awareness of emergency contraceptives among women of reproductive age in sub-Saharan Africa: a scoping review

**DOI:** 10.1186/s40834-022-00167-y

**Published:** 2022-01-17

**Authors:** Kelvin Amaniampong Kwame, Luchuo Engelbert Bain, Emmanuel Manu, Elvis Enowbeyang Tarkang

**Affiliations:** 1 School of Public Health, University of Health and Allied Sciences, PMB 31 Ho, Ghana; 2 Lincoln International Institute for Rural Health, LIIRH, University of Lincoln, Lincoln, UK; 3 Global South Health Services and Research, GSHS, Amsterdam, The Netherlands; 4 HIV/AIDS Prevention Research Network Cameroon, PO Box 36, Kumba, Cameroon

**Keywords:** Emergency contraception, Women, Sub – Saharan Africa

## Abstract

**Background:**

Emergency contraception (EC) is a method used to avoid pregnancy after unprotected sexual intercourse. Emergency contraceptives can reduce the risk of unintended pregnancy by up to over 95% when taken within 72 h of sexual intercourse. EC is helpful to women who have experienced method failure, incorrect use of contraceptives, raped or have consented to unplanned, and unprotected sexual intercourse. We set out to systematically review the current literature on the awareness and usage patterns of ECs among women of reproductive age in Sub-Saharan Africa.

**Method:**

Eight hundred and sixty-seven (867) articles were selected from EMBASE and Google Scholar databases after a search was conducted. Sixty (60) full-text articles were checked for eligibility and 27 articles met our inclusion criteria. Manual data extraction on excel sheets was used to extract the authors’ names, year of publication, country, sample size, study type, objectives, awareness levels, and the EC types.

**Findings:**

Awareness rates ranged from 10.1 to 93.5% (both reported from Ethiopia). The level of use was relatively low (ranging from 0% in DR Congo and Ethiopia to 54.1% in Nigeria). The most used types of EC were Postinor 2 (levonorgestrel), EC pills such as Norlevo (levonorgestrel only) and Nodette (levonorgestrel and estradiol), and intrauterine contraceptive device (IUCD).

**Conclusions:**

Although variations in use and awareness do exist between countries in SSA according to the year of study, the general level of EC awareness has been on the increase. On the other hand, the level of EC use was lower compared to the level of awareness. Postinor 2 (levonorgestrel-only pills) was reported as the most type used EC. Further, studies could be done to find out the effect of culture, religion and believes on the use of contraceptive methods. It is important to understanding barriers to EC use despite high awareness rates. Emergency Contraceptive awareness and use should be promoted among women of reproductive age in SSA to reduce unwanted pregnancies and their complications.

## Introduction

The prevalence of unwanted pregnancy and abortion continues to rise in sub-Saharan Africa. Unintended pregnancies remain an important public health issue. Globally, 74 million women living in low and middle-income countries (LMICs) have unintended pregnancies annually. This leads to 25 million unsafe abortions and 47,000 maternal deaths every year [1]. Unintended pregnancy and unsafe abortion can be prevented by the use of contraceptive methods including emergency contraceptives. Emergency contraceptives (EC) is a method used to avoid pregnancy after unprotected sexual intercourse. It is used before the potential time of implantation, unlike the regular contraceptive methods that are administered before sexual intercourse. Emergency contraceptives can reduce the risk of unintended pregnancy by up to over 95% when taken within 72 h of sexual intercourse [2]. The use of modern contraceptives in 2017 prevented an estimated 308 million unintended pregnancies [3]. Meeting all women’s need for modern methods of contraception would avert an additional 67 million unintended pregnancies annually [3]. Despite the benefits of contraceptives, their use in Africa is 29.4% among women between the ages of 15 and 49 years despite high rates of maternal mortality in the continent [4]. Amongst the global regions, SSA has the lowest contraceptive prevalence rate (CPR) of 29% [5]. The SSA region experiences more than 14 million unplanned pregnancies each year with an overall prevalence rate of 29% [6]. About 44% of all pregnancies worldwide are unintended, and some 56% of unintended pregnancies end in induced abortion. An estimated 56 million induced abortions took place annually in 2010–2014, which translates to an annual abortion rate of 35 for every 1000 women aged 15–44 years [7]. Sub-Saharan Africa constitutes roughly 66% of the world’s maternal deaths [8].

Emergency contraceptives provide women of reproductive age with an opportunity to prevent an unplanned or mistimed pregnancy within three to five days of unprotected sexual intercourse by preventing or temporarily stopping ovulation or by causing a chemical change in sperm and egg before they meet [2]. However, despite the availability, safety, and efficacy of the specific emergency contraceptive agents, there is still limited awareness and use of EC among women of reproductive age in SSA [9].

Contraceptive use improves women’s and children’s health in many ways, including reducing maternal mortality risks, and improving child survival through birth spacing and the nutritional status of both mothers and children [8]. The use of modern contraceptive methods such as EC could prevent the majority of abortions and many maternal deaths [3]. In developed regions, it is estimated that 30 women die for every 100,000 unsafe abortions. That number rose to 220 deaths per 100,000 unsafe abortions in developing regions and 520 deaths per 100,000 unsafe abortions in SSA. Mortality from unsafe abortion disproportionately affects women in Africa. While the continent accounts for 29% of all unsafe abortions, it sees 62% of unsafe abortion-related deaths [10]. Sub-Saharan African countries have a high rate of unintended pregnancies due to inadequate access to women’s reproductive health services [11]. This implies that most SSA countries have limited access to facilities for family planning and reproductive health rights, which in a way may not contribute to achieving the UN Sustainable Development Goal 3 (SDG 3): improving maternal health, reducing maternal mortality, and achieving universal access to reproductive health [12]. Despite several engagements to reduce or eradicate mother and child deaths globally through the century, the health consequences of unplanned pregnancies are a significant public health concern, particularly for women residing in developing countries [11].

Each year between 4.7 and 13.2% of maternal deaths are attributed to unsafe abortion. The incidence of unsafe abortion stands at 25 million annually. Three (3) out of four (4) abortions that occur in Africa are unsafe and the risk of dying from an unsafe abortion is highest in Africa [10]. A total of 295,000 women worldwide lost their lives during and following pregnancy and childbirth in 2017, with SSA and South Asia accounting for approximately 86% of all maternal deaths worldwide [8]. The adverse effects of pregnancy on maternal health that can be avoided by the use of EC are experienced mostly in SSA. Emergency Contraceptives can prevent unsafe abortions and reduce maternal mortality by reducing the number of unintended pregnancies. The limited awareness and use of EC even in situations of potential regular contraceptive failure is a public health problem in SSA. This study seeks to review and map the available evidence on the level of awareness and usage of EC among women of reproductive age in SSA and summarize available evidence to provide an overview of EC awareness and use in SSA.

## Methodology

This study is a scoping review of published articles on the awareness and use of EC among women of reproductive age in SSA. The methodological framework used is according to the five-stage layout process described by Arksey and O’Malley (2005) [13]. This method follows a five-stage process. Stage one (1) involves identifying a research question; stage two (2) involves identifying relevant studies; stage three (3) involves the study selection; stage four (4) involves charting of the data and stage five (5) involves collating, summarizing and reporting the results.

### Search strategy

All citations were imported into the web-based bibliographic manager Mendeley Desktop Version 1.19.4 by Glyph & Cog, LLC (Elsevier Database), and duplicate citations were removed manually with further duplicates removed when found later in the process. A systematic search was conducted in EMBASE and Google Scholar for articles that met the eligibility criteria by the authors (A.K., E.T., & M.E). The search followed the Preferred Reporting Items for Systematic Reviews and Meta-Analyses (PRISMA) guidelines. The following terms were used (including synonyms and closely related words) as index terms or free-text words: “Sub Saharan Africa”, “women”, “Family Planning”, “Contraceptives”, “Birth Control”, “female”,“emergency”, “awareness”, “Knowledge”, “Practice”, and “Use”. Two reviewers independently conducted abstract screening followed by a full-article screening of selected studies, using standardized tools, with guidance from the eligibility criteria. When there were disputes, a third reviewer decided on the course of action.

The inclusion criteria used for this review were; studies that presented evidence that was published from 2000 to 2019, published on sub-Saharan Africa, and also presented evidence on emergency contraceptive awareness and use among women aged 15–49 years. The following exclusion criteria were used during this review; Articles published in languages other than English without translation, studies published with information on only one of the specific objectives (either awareness or use), and titles for which full text could not be retrieved. The search was performed with English language restriction. The date was restricted to 2000–2019.

### Data extraction

The three authors participated in the article selection and data extraction processes. All citations deemed relevant after the title and abstract screening were downloaded for subsequent review of the full-text articles. A data charting table was used to extract background information and process the information from each utilized study. The data extraction form was developed, piloted, and used to extract and process relevant information from each included study by the authors (A.K., E.T., & M.E). All articles reviewed were assigned a unique code to help track them. The form captured information on: Author(s), year of publication, origin/country of origin (where the study was published or conducted), aim/purpose, sample size (if applicable), methodology (study design), and key findings that relate to the scoping review question(s).

## Results

### Summary of findings

The search conducted on in PubMed and Google Scholar, yielded 856 potentially relevant citations. After duplication removal and relevant screening, 60 citations met the eligibility criteria based on title and abstract. The corresponding full-text articles were downloaded for review. After the characterization based on the objectives of the study, 33 full-text articles were excluded from the scoping review.

Three sample size categories (below 500, 500–2000, & above 2000) were used to classify the sample sizes of all the included review articles. The majority (63%)of articles included in this review had a sample size of below 500. The minimum sample size was 32 participants from a qualitative study conducted in Ghana [14] whiles the maximum sample size was 7785 (Kenyans) plus 12,487 (Nigerians) from cross-sectional studies conducted in these two countries simultaneously [15]. The articles included in this review were published from 2003 [16] to 2019 [17]. In all, five (5) studies were conducted in Ghana, eight (8) in Nigeria, one (1) in DR Congo, three (3) in South Africa, one (1) in Swaziland, two (2) in Kenya, seven (7) in Ethiopia, one (1) in Cameroon, and one (1) in Senegal. Two of these final twenty-seven selected articles were conducted on two countries simultaneously; Kenya and Nigeria [15, 18].

In all, 18.5% of the articles were from Ghana, 22.2% from Nigeria, 26.0% from Ethiopia, 11.1% from South Africa, 7.4% from Nigeria and Kenya, and 3.7% from DR Congo, Swaziland, Senegal, and Cameroon each. The review had no limits on the study design; so, articles with varied designs were included in the study. The majority (88.9%) of the study designs were cross-sectional. Other designs included mixed-method and qualitative. Some reviews were conducted as stand-alone projects while others were undertaken as parts of larger research projects. The general characteristics of scoping reviews included in this study are reported in Table 1. The summary of the findings are presented in Table 2.

**Table 1:** Study characteristics of included review studies

Characteristic	Frequency (***n*** = 27)	Percentage (%)
**Country of Publication**		
Ethiopia	7	26.0
Ghana	5	18.5
Nigeria	6	22.2
Kenya & Nigeria	2	7.4
South Africa	3	11.1
DR Congo	1	3.7
Cameroon	1	3.7
Swaziland	1	3.7
Senegal	1	3.7
**Sample Size Range**		
Below 500	17	63
500–2000	6	22.2
Above 2000	4	14.8
**Study Design**		
Cross–sectional	24	88.9
Mixed method	1	3.7
Qualitative	2	7.4

**Table 2:** Summary of findings from included studies

Author	Country	Study design	Results
**Aziken, Okonta & Ande, (2003)** **[**16**]**	Nigeria	Cross-sectional study	**Awareness:** 510 women (58%) were aware of EC **Use:** 2.1% has ever used EC **Type(s):** Combined oral contraceptives, Dedicated levonorgestrel-only pills, Menstrogen, Brown codeine, Ampicillin, Quinine, Ergometrine & Gynaecosid
**Mqhayi et al, (2004)** **[**19**]**	South Africa	Cross-sectional study	**Awareness:** 32 **(**16.6%) knew about EC **Use:** 2 (1.03%) had ever used EC **Type(s):** All
**Ikeme, Ezegwui & Uzodimma** **(2005)** **[**20**]**	Nigeria	Quantitative study	**Awareness:** 256 (61%) were aware of EC **Use:** 31% had used EC **Type(s):** Postinor
**Ebuehi, Ekanem & Ebuehi, (2006)** **[**21**]**	Nigeria	Cross-sectional descriptive study	**Awareness:** 320 **(**67.8%) knew about EC **Use:** 62 **(**33.9%) out of 183 having ever practiced EC (12.9% of the sample) **Type(s):** Menstrogen, Postinor 2, combined oral contraceptives, Levonorgestrel & Noriday
**Myer et al, (2007)** **[**22**]**	South Africa	Cross-sectional study	**Awareness:** Out of 831, 253 (30%) of the women had ever heard of EC **Use:** 13% of the 253 (those aware of EC); 4% of the entire participants (831) used EC (*n* = 34) **Type(s):** All
**Kongnyuy et al, (2007)** **[**23**]**	Cameroon	Cross-sectional study	**Awareness:** General level of awareness of EC was 63.0% (418/664) **Use:** 49 (7.4%) had used EC **Type(s):** All
**Addo & Tagoe-Darko, (2009)** **[**24**]**	Ghana	Cross-Sectional Study	**Awareness:** 1178 (51.4%) of the 2292 respondents reported having heard about Emergency Contraceptives **Use:** 96 (4.2%) respondents had ever used Emergency Contraceptives **Type(s):** N-tablets, contraceptive Pills, Postinor, and an IUCD.
**Tilahun, Assefa & Belachew, (2010)** **[**25**]**	Ethiopia	Cross-sectional study	**Awareness:** out of 310**,** 62 (20%) respondents were aware of EC **Use:** out of the respondents, 62 had ever used emergency contraceptives (9.4%) **Type(s):** ECPs, IUCD and some didn’t remember the type.
**Opoku & Kwaununu, (2011)** **[**26**]**	Ghana	Cross-sectional study	**Awareness:** Out of 402 respondents, 229 (57%) knew about EC **Use:** 163 (71% of those aware of EC) had used it before. This formed 41% of all participants that responded (402). **Type(s):** All
**Ahmed et al, (2012)** **[**27**]**	Ethiopia	Cross-sectional study	**Awareness:** 310 (84.2%) had ever heard of EC **Use:** Out of 368, 7.3% had ever used EC (75% of sexually active respondent) **Type(s):** All
**Hoque & Ghuman, (2012)** **[**28**]**	South Africa	Cross-sectional study	**Awareness:** 49.8% of the participants reported having heard about EC **Use:** Out of 453 sexually active students, (21.2%) used EC (11.3% of the sample). **Type(s):** Norlevo, Ovral, Microval, Nordette, IUCD
**Tesfaye, Tilahun & Girma, (2012)** **[**29**]**	Ethiopia	Cross-Sectional Study	**Awareness:** Out of the 89 respondents, 9 (10.1%) knew about EC **Use:** None of the respondents used EC **Type(s):** progesterone only pills & IUCD
**Abate, Assefa & Alemayehu, (2014)** **[**30**]**	Ethiopia	Cross-sectional study	**Awareness:** 162 women (41.5%) heard about EC **Use:** 38 (9.7%) used EC **Type(s):** All
**Amalba, et al, (2014)** **[**31**]**	Ghana	Cross-sectional study	**Awareness:** Awareness level of ECPs were found to be 69.0% (138) **Use:** 55 (39.9%) of the participants who had awareness have ever used ECPs (27.5% of participants) **Type(s):** All
**Chin-Quee et al, (2014)** **[**18**]**	Kenya & Nigeria	Cross-sectional study	**Awareness:** 2396 (79%) in Nairobi and 2065 (66%) in Lagos had heard of ECPs **Use:** 546 (18%) from Nairobi and 531 (17%) in Lagos had ever ECPs **Type(s):** All
**Morgan, Keesbury &Speizer, (2014)** [15]	Kenya & Nigeria	Cross-sectional study	**Awareness:** Kenya 4486 (58%) and 3890 (31%) in Nigeria were aware of EC **Use:** 856 (11%) in Kenya, 786 (6.3%) in Nigeria. **Type(s):** All
**Nibabe & Mgutshini, (2014)** **[**32**]**	Ethiopia	Cross-sectional study	**Awareness**: 246 (69.9%) of the respondents knew about EC **Use:** Out of 352 respondents, 38 (10.8%) admitted to ever having used EC (i.e.; 15.4% of 246 who were aware of EC) **Type(s):** All
**Yam et al, (2014)** **[**33**]**	Swaziland	Cross-sectional study	**Awareness:** 27.5% were aware of EC **Use:** 27.5% of respondents had ever used ECP **Type(s):** All
**Mane et al, (2015)** **[**34**]**	Senegal	Mixed-Method (Interviews and Surveys)	**Awareness:** 20% were aware of EC **Use:** 4% had ever used EC **Type(s):** All
**Shiferaw, Gashaw & Tesso, (2015)** **[**35**]**	Ethiopia	Cross-sectional study	**Awareness:** Out of 489, 332 (67.8%) respondents had heard of EC **Use:** 68 (36.2%) used EC (13.9% of sample) **Type(s**): ECPs & IUCD
**Abiodun, (2016)** **[**36**]**	Nigeria	Cross-sectional study	**Awareness:** 964 (72.6%) were aware of emergency contraceptives **Use:** 718 (54.1%) had ever used emergency contraceptives. **Type(s):** All
**Onasoga et al, (2016)** **[**37**]**	Nigeria	Cross-sectional study	**Awareness:** 173 (86.5%) of respondents have heard of emergency contraceptive pills **Use:** Out of 200 respondents, 61 (30.5%) have used emergency contraceptive **Type(s):** Postinor-2 & Don’t know
**Ajayi et al, (2017)** **[**38**]**	Nigeria	Cross-sectional study	**Awareness:** Out of 370 respondents, 63.1% were aware of EC **Use:** 27.4% out of 330 used EC **Type(s):** Levonorgestrel (postinor) & non-EC. **Non-EC** drugs reported by the participants include: menstrogen, gynacocied, antibiotics, Cytotec, Andrews liver salt, MNB 760, Alabukun, salt and water, alcohol, lime, potash, and yoyo bitters
**Hernandez et al, (2017)** **[**39**]**	DR Congo	Qualitative Study; Phenomenology	**Awareness:** very few participants reported having heard of EC **Use:** No participant used EC **Type(s):** douching, drinking salted water or sodas, using an herbal concoction or even jumping hard, antibiotics, deworming medicines (Décaris, Tanzol), and antimalarial medicines (quinine, tetracycline) what were used as after sex methods.
**Rokicki & Merten, (2018)** **[**14**]**	Ghana	Qualitative Study; Phenomenology	**Awareness:** Awareness was high amongst the respondents, 26 out of 32. **Use:** Twenty-six of the 32 participants had used ECPs at least once. **Type(s):** Postinor-2.
**Mishore, Woldemariam & Huluka, (2019)** **[**40**]**	Ethiopia	Cross-sectional study	**Awareness:** Out of 214 respondents, 200 (93.5%) were aware of EC. **Use:** Out of 200 who were aware, 66 (33%) used EC (30.8% of respondents) **Type(s):** Pills, IUCD & Implants and Injectable
**Mohammed, Abdulai & Iddrisu, (2019)** **[**17**]**	Ghana	Cross-sectional study	**Awareness:** 166 (86.91%), of the participants, indicated they had heard about emergency contraceptives **Use:** 49 (25.7%) participants used EC **Type(s):** IUCD

#### Awareness of emergency contraceptives among women of reproductive age in sub-Saharan Africa

Awareness of EC among women of reproductive age in SSA ranged from 10.1% in a study conducted by Tesfaye, Tilahun, and Girma (2012) [29] to 93.5% in a study conducted by Mishore, Woldemariam, and Huluka (2019) [40]. These two studies were both conducted in Ethiopia. Even though the study settings and participants differed, some countries recorded a higher level of awareness on EC than others (Table 3).

**Table 3:** Awareness of emergency contraceptives among participants from selected studies

Study	***EC Awareness (%)***
*Aziken, Okonta & Ande, 2003*	**58**
*Mqhayi* et al*, 2004*	**16.6**
*Ikeme, Ezegwui & Uzodimma, 2005*	**61**
*Ebuehi, Ekanem & Ebuehi, 2006*	**67.8**
*Myer* et al*, 2007*	**30**
*Kongnyuy* et al*, 2007*	**63**
*Addo & Tagoe-Darko, 2009*	**51.4**
*Tilahun, Assefa & Belachew 2010*	**20**
*Opoku & Kwaununu, 2011*	**57**
*Ahmed* et al*, 2012*	**84.2**
*Hoque & Ghuman, 2012*	**49.8**
*Tesfaye, Tilahun & Girma, 2012*	**10.1**
*Abate, Assefa & Alemayehu 2014*	**41.5**
*Amalba* et al*, 2014*	**69.0**
*Chin-Quee* et al*, 2014*	**79 (K) & 66 (N)**
*Morgan, Keesbury &Speizer, 2014*	**58 (K) & 31 (N)**
Nibabe & Mgutshini, 2014	**69.9**
*Yam* et al*, 2014*	**27.5**
*Mane* et al*, 2015*	**20**
*Shiferaw, Gashaw & Tesso, 2015*	**67.8**
*Abiodun, 2016*	**72.6**
*Onasoga* et al*, 2016*	**86.5**
*Hernandez* et al*, 2017*	**Very few #**
*Ajayi* et al*, 2017*	**63.1**
*Rokicki & Merten, 2018*	**81.3 #**
*Mishore, Woldemariam & Huluka, 2019*	**93.5**
*Mohammed, Abdulai & Iddrisu, 2019*	**86.9**

# Qualitative studies, K = Kenya, N = Nigeria.

**Fig. 1:**
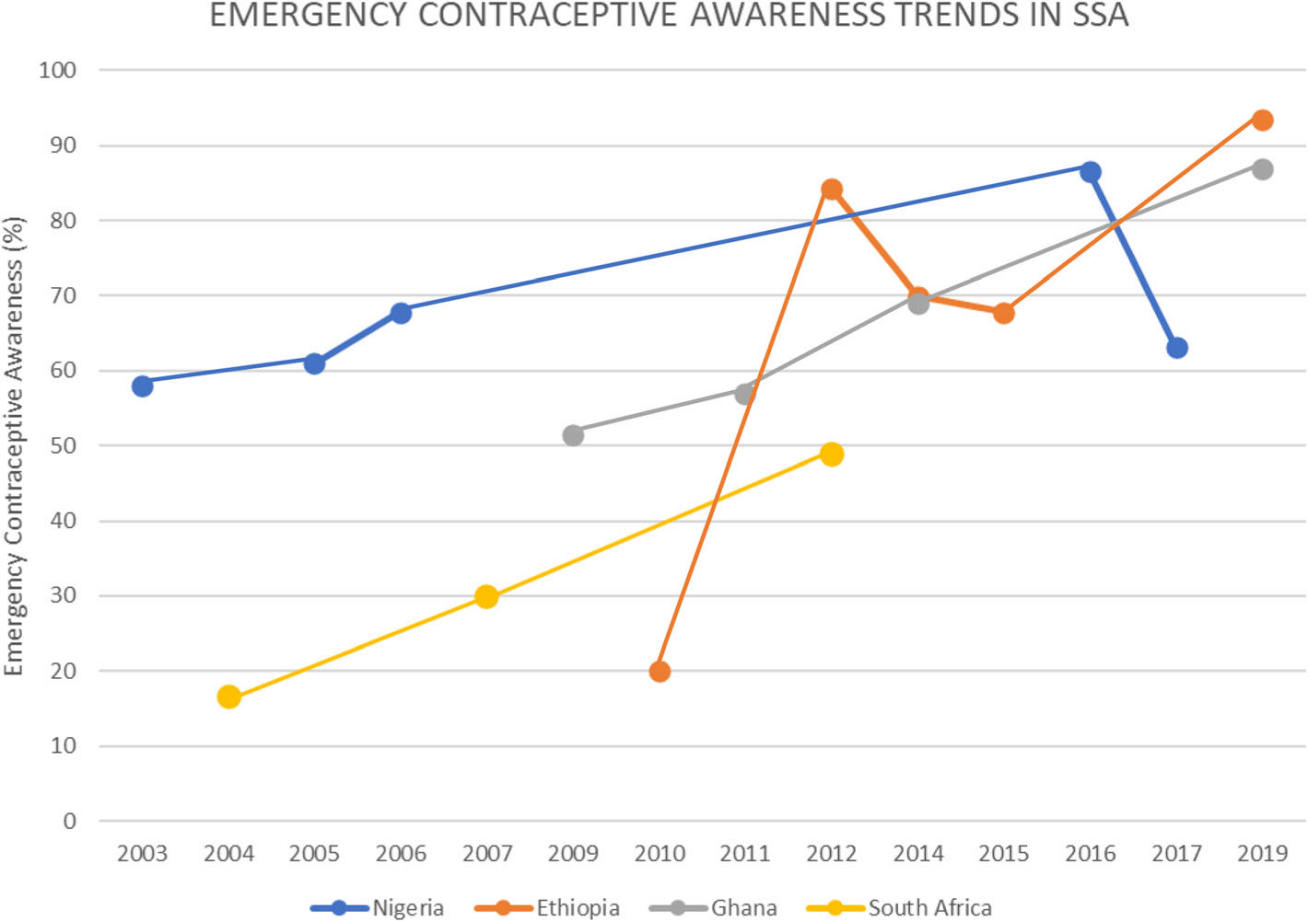
Emergency Contraceptive Awareness Trends in four sub-Saharan countries

When three or more studies were reported on EC awareness on a country for different years, a trend analysis was done to examine the pattern of EC awareness (Fig. 1). A study from Nigeria conducted in the year 2003, reported 58% of 880 participants of a study were aware of EC [16]. In the years that followed, which were 2005 (61%) [20] and 2006 (67.8%) [21] were reported from other studies carried out in the same country. 72.6% level of EC awareness was reported in the year 2016 [36] which was also conducted in Nigeria. These findings indicate that the level of awareness on EC in Nigeria increased over the years. In 2017, the level of awareness reported from a cross-sectional study conducted [38] was lower (63.1%) compared to that of a study conducted in the previous year (86.5%) [37]. Figure 1 shows the awareness of EC from Nigerian studies included in the review.

Three studies were selected from South Africa, which were published in the years 2004, 2007, and 2012. South Africa recorded an increase in the level of awareness from 16.6% in 2004 [19] to 49.8% in 2012 [28], though the study regions varied for all the three studies.

Two comparative studies were conducted in the year 2014 between Kenya and Nigeria on EC. The first study sampled 3033 and 3129 participants from Kenya and Nigeria respectively [18]. The second study sampled 7785 (Kenyans) and 12,487 (Nigerian) participants [15]. The reported level of awareness for the two studies were 79 and 58% in Kenya, while Nigeria recorded 66 and 31% [15, 18].

A study conducted in Ethiopia by Tilahun, Assefa, and Belachew (2010) [25], recorded a level of awareness of 20% of the 660 study participants. From the year 2012, two studies conducted in Ethiopia met the current scoping review’s inclusive criteria. This year recorded the lowest level of awareness of 10.1% [29] from all the selected articles combined. In the same year, another study was conducted in a different region of Ethiopia and reported an awareness level of 84.2% [27]. About 41.5 and 69.9% awareness level were recorded from two studies from different regions in Ethiopia in the year 2014 [30, 32]. In 2015 and 2019, other studies reported a level of EC awareness as 67.8% [34] and 93.5% [40].

A study in Ghana recorded a level of awareness of 51.4% in the year 2009 [24]. Two years down the line, another study from Ghana recorded an increase in the level of awareness to 57% [26]. A study conducted by Amalba et al. (2014) [31] in Ghana recorded a level of awareness of 69.0%. whiles a cross-sectional study conducted in Ghana in the year 2019 recorded a level of awareness of 86.9% [17]. A qualitative study was conducted in Ghana with 32 participants of which the majority (26) of the participants were aware of EC [14]. Figure 1 shows the trend of increase of the level awareness in Ghana over the years from the included articles.

A 63% level of awareness from 664 respondents was recorded from a study conducted in Cameroon to evaluate the knowledge, attitudes, and experiences on EC pills by the university students [23]. A study to examine EC use among female sex workers in Swaziland sampled 325 participants and reported that 27.5% of these workers aware of EC [33]. A cross-sectional study conducted in Senegal reported the level of EC awareness to be 20% out of 9614 study participants [34]. A study conducted in DR Congo was qualitative hence inference could not be made to the population. The number of participants that were aware of EC was not clearly stated because the study reported that few participants were aware of EC [39].

### Use of emergency contraceptives among women of reproductive age in sub-Saharan Africa

Use of EC among women of reproductive age in SSA reported by the various studies included in this review ranged from as low as 0% [29] to as high as 541% [36] from Ethiopia and Nigeria respectively. The two qualitative studies reported non-use of EC from DR Congo [39], and 81.3% use from Ghana [14]. Even though the study settings and participants differed, some countries recorded use of EC above 30% [20, 26, 36, 37, 40]. Table 4 shows the level of EC use extracted from the selected articles used for the review.

**Table 4:** Level of Emergency Contraceptive use from selected studies

Study	***EC use (%)***
*Aziken, Okonta & Ande, 2003*	**2.1**
*Mqhayi* et al*, 2004*	**1.03**
*Ikeme, Ezegwui & Uzodimma, 2005*	**31**
*Ebuehi, Ekanem & Ebuehi, 2006*	**12.9**
*Myer* et al*, 2007*	**4**
*Kongnyuy* et al*, 2007*	**7.4**
*Addo & Tagoe-Darko, 2009*	**4.2**
*Tilahun, Assefa & Belachew 2010*	**9.4**
*Opoku & Kwaununu, 2011*	**41**
*Ahmed* et al*, 2012*	**7.3**
*Hoque & Ghuman, 2012*	**11.3**
*Tesfaye, Tilahun & Girma, 2012*	**0**
*Abate, Assefa & Alemayehu 2014*	**9.7**
*Amalba* et al*, 2014*	**27.5**
*Chin-Quee* et al*, 2014*	**18 (K) & 17 (N)**
*Morgan, Keesbury &Speizer, 2014*	**11 (K) & 6.3 (N)**
Nibabe & Mgutshini, 2014	**10.8**
*Yam* et al*, 2014*	**27.5**
*Mane* et al*, 2015*	**4**
*Shiferaw, Gashaw & Tesso, 2015*	**13.9**
*Abiodun, 2016*	**54.1**
*Onasoga* et al*, 2016*	**30.5**
*Hernandez* et al*, 2017*	**0 #**
*Ajayi* et al*, 2017*	**27.4**
*Rokicki & Merten, 2018*	**81.3 #**
*Mishore, Woldemariam & Huluka, 2019*	**30.8**
*Mohammed, Abdulai & Iddrisu, 2019*	**25.7**

# Qualitative studies, K = Kenya, N = Nigeria.

**Fig. 2:**
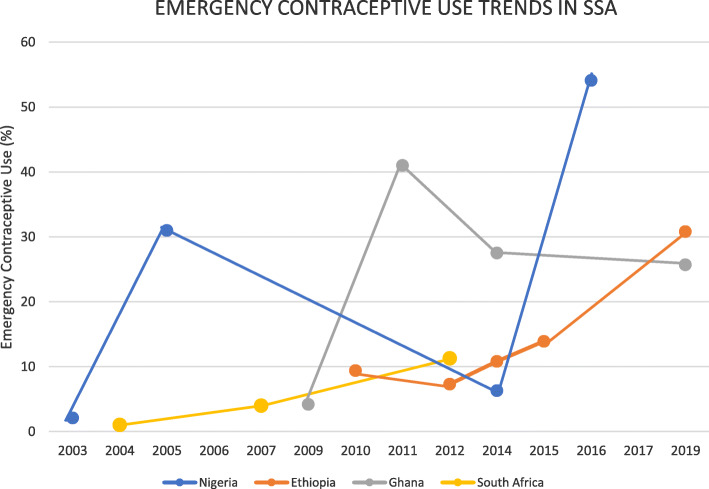
Emergency Contraceptive Use Trends in four sub-Saharan countries

When three or more studies were reported on EC use on a country for different years, a trend analysis was done to examine the pattern of EC use (Fig. 2). A qualitative study conducted in Ghana reported 32 of 36 (81.3%) participants to use EC [14]. The lowest level of EC use reported from Ghana from the selected review studies was 4.2% [24], while the highest was 41% in the year 2011 [26]. After the year 2011, Ghana experienced a decreasing trend [17, 31] (Fig. 2).

Three studies from Nigeria recorded over a 30% level of EC use [20, 36, 37]. The highest level of EC use in Nigeria from the selected studies was 54.1% which was also the highest recorded from the included review articles This study sampled 1328 participants and 718 reported to have used EC [36]. Compared to the level of awareness recorded in this same study, the level of use was relatively lower. Nigeria recorded the lowest level of EC use of 2.3% in 2003 [16], which increased to 31% in the year 2005 [20]. In the year that followed, a study in Nigeria reported a drop to 12.9% [21]. Two studies conducted in 2014 from Nigeria reported the level of EC use of 6.3% [15] and 17% [18]. Two years later, two studies included in this review from Nigeria reported the levels of EC use of 54.1% [36] and 30.4% [37]. Figure 2 shows the trend of EC use in Nigeria over the years from the included articles. The highest level of EC use recorded in Ethiopia from the selected studies was 30.8% [40]. In the year 2012, another study from Ethiopia that was included in this review recorded 0% EC use [29]. In the year 2014, two studies conducted in Ethiopia reported levels of EC use of 9.7% [30] and 10.8% [32] (Fig. 2).

### Types of emergency contraceptives used among women of reproductive age in sub-Saharan Africa

Concerning the types of EC used, some of the selected studies did not report on any specific type of EC: these studies reported on “All” types of EC. About 13 out of the 27 articles reported on “All” EC since they were not specific as to the type they were reporting on [15, 18, 19, 22, 23, 26, 27, 30–34, 36]. Some selected articles for the review reported on several types of EC and some other non-EC methods that were used. The non-EC types used were: Menstrogen, Brown codeine, Ampicillin, Quinine, Ergometrine, Gynaecosid, antibiotics, and Cytotec [16, 21, 38]. All the studies that reported non-EC methods were conducted in Nigeria. It was reported in these studies that normal contraceptives were used sometimes as EC by increasing the dosage. A study from Nigeria also reported the likes of Andrews liver salt, thiazamide (MNB 760), Alabukun (local aspirin), salt and water, alcohol, lime, potash, and yoyo bitters being used as EC [38]. Table 5 contains a summary of the types of EC used by women of reproductive age in SSA.

**Table 5:** Types of Emergency Contraceptives used by women of reproductive age in Sub-Saharan Africa

Study	Types of EC use
*Aziken, Okonta & Ande, 2003*	Combined oral contraceptives, Dedicated levonorgestrel-only pills, Menstrogen, Brown codeine, Ampicillin, Quinine, Ergometrine & Gynaecosid
*Mqhayi* et al*, 2004*	All
*Ikeme, Ezegwui & Uzodimma, 2005*	Postinor
*Ebuehi, Ekanem & Ebuehi, 2006*	Menstrogen, Postinor, combined oral contraceptives, Levonorgestrel & Noriday
*Myer* et al*, 2007*	All
*Kongnyuy* et al*, 2007*	All
*Addo & Tagoe-Darko, 2009*	N-tablets, contraceptive Pills, Postinor, and an IUCD
*Tilahun, Assefa & Belachew 2010*	ECPs, IUCD & didn’t remember the type.
*Opoku & Kwaununu, 2011*	All
*Ahmed* et al*, 2012*	All
*Hoque & Ghuman, 2012*	Norlevo, Ovral, Microval, Nordette, IUCD
*Tesfaye, Tilahun & Girma, 2012*	Progesterone only pills & IUCD
*Abate, Assefa & Alemayehu 2014*	All
*Amalba* et al*, 2014*	All
*Chin-Quee* et al*, 2014*	All
*Morgan, Keesbury &Speizer, 2014*	All
Nibabe & Mgutshini, 2014	All
*Yam* et al*, 2014*	All
*Mane* et al*, 2015*	All
*Shiferaw, Gashaw & Tesso, 2015*	Emergency Contraceptive Pills & IUCD
*Abiodun, 2016*	All
*Onasoga* et al*, 2016*	Postinor-2 & Don’t know
*Hernandez* et al*, 2017 #*	Douching, drinking salted water or sodas, using herbal concoction or even jumping hard, antibiotics, deworming medicines (Décaris, Tanzol) & antimalarial medicines (quinine, tetracycline)
*Ajayi* et al*, 2017*	Postinor. Non-EC drugs reported: menstrogen, gynacocied, antibiotics, Cytotec, Andrews liver salt, MNB 760, Alabukun, salt and water, alcohol, lime, potash, and yoyo bitters
*Rokicki & Merten, 2018 #*	Postinor 2
*Mishore, Woldemariam & Huluka, 2019*	Pills, IUCD & Implants and Injectable
*Mohammed, Abdulai & Iddrisu, 2019*	IUCD

# = Qualitative studies

The most commonly used EC types from all the studies in SSA used in this review were: **Postinor 2** [14, 20, 21, 24, 37, 38], **Emergency contraceptive pills** [2, 16, 25, 28, 29, 35, 40], and **IUCDs** [17, 24, 25, 28, 29, 35, 40]. Few of the selected studies reported that participants did not know the type of EC that was used or could not remember at the time of the study [25, 37].

## Discussion

### Awareness of emergency contraceptives among women of reproductive age in sub-Saharan Africa

The awareness of ECs varies across countries, with the highest awareness rates of 93.5% recorded in Ethiopia [40].

Generally, the level of awareness on EC among women of reproductive age in SSA has been on the rise. A study by Robert et al. (2004) [41] from South Africa reported the level of EC awareness to be 57%, which is higher than that recorded by Hoque and Ghuman (2012) [28]. The reported level of EC awareness from the other selected studies conducted in South Africa were 16.6% [19], 30% [22], and 49.8% [28]. These differences could be accounted for the differences in specific questions asked to capture awareness levels regarding EC, as well as the target population. Awareness of EC among university students in Ethiopia, who are believed to have better knowledge and awareness of EC were below 50% [42–45]. This disconnect between high levels of education and low awareness rates could be explained by lack of EC education in comprehensive education package, as well as sub – optimal health education from the health care system. Concerning the age distribution of the study participants from the various included study articles of this review, 18 out of 27 articles reported the majority of their participants were between the ages of 20–24 years [14, 16, 19–26, 28, 30, 33, 35, 36, 38, 39]. This was followed by the age group of over 24 which was reported as the majority of their participants for 5 out of the 27 included review articles [15, 17, 18, 22, 34]. The remaining articles (4 out of 27) reported below 20 years as the majority of their study participants signifying the adolescent group [27, 29, 37, 40]. Based on the majority of the study participants of all the included articles in this review, it suggests that the level of EC awareness was higher in 20–24 years age group compared to that of above 24 years. This could be as a result of the 20-24 years age group having more access tertiary institution that can make information on the method readily available to them rather than the adolescent age group (15–19 years). Also the African society frowns on sexual activities by the young adult, therefore there is an inherent limit on the amount of information an adolescent can get on contraception including ECs [46].

### Use of emergency contraceptives among women of reproductive age in sub-Saharan Africa

Emergency contraceptive pills (ECPs) are now available in many countries but have failed to have the desired impact on unwanted pregnancy rates in the world especially in Africa [47]. Earlier barriers of access to EC are becoming less and less prevalent [48]. Emergency contraceptives is an essential, although often underutilized family planning option in most parts of the world including SSA. In SSA, where access to formal health care and family planning services remains limited, ECs, which are often accessed through private sector pharmacies have emerged to play an important role in preventing unwanted pregnancies. Evidence from this review is contrary to claims in some settings that the use of EC is widespread. For instance, media reports of overuse in Nigeria and Kenya are not supported by the relatively low levels of use found in these countries compared to the high level of EC awareness [15, 16, 18, 21, 38]. The findings of this study demonstrate that the trends of EC use differed from patterns of awareness. The findings from this review are in line with the report from the UN, which states that Africa has a low use of contraceptives including EC [49].

The low use of EC over the years can be a result of population increase, decreased access to services, or discontinuation of the method by users. Further studies need to be conducted to ascertain and understand the low use of EC despite its increased awareness among women of reproductive age in SSA. There was a relatively lower use of EC compared to the level of EC awareness in all the included review studies. This gives a clear indication that knowing or being aware of the contraception methods is not the only factor necessary or responsible for their use. Factors that influence the uptake or use of EC can be explored to help understand the reasons for the low level of EC use. Sub-Saharan Africa is a region with a strong culture and norms. This can be the reason why the use of EC is still generally low. The generally low level of EC use among women of reproductive age in SSA can be a reason why unintended pregnancies in Africa as a whole is still high. People in SSA have high levels of awareness of EC but relatively lower usage.

With the majority of study participants from the included review studies being in the 20–24 years age group, it also suggests that EC users in SSA are predominantly found in this age group more than adolescents and people over 24. This could be as a result that, people over 24 years might be married and hence might not need to use ECs as much as the 20–24 years. This same age group (over 24 years) are more matured and hence might practice safe sex. Adolescent age group on the other hand, might be sexually active but might lack the funds to acquire an EC on the market [46].

### Types of emergency contraceptives used among women of reproductive age in sub-Saharan Africa

The review found out that some studies reported that normal contraceptives were used sometimes as EC by increasing the dosage. A worrisome observation was the wrong identification of Menstrogen and Gynaecosid as EC in some of the included review studies [16, 21, 38]. Menstrogen, which is an Oestrogen-only pill used in the treatment of conditions related to low hormonal levels was reported by studies as a used type of EC [16, 21, 38]**.** Gynaecosid, which is recommended for the treatment of amenorrhoea was reported to be used as EC [16, 38]. These included review studies that reported the use of Menstrogen and Gynaecosid as EC were all from Nigeria [16, 21, 38]. The effectiveness of Menstrogen and Gynaecosid when used as an emergency contraceptive requires use in high doses and hence harmful to the health of the user. Also, Menstrogen and Gynaecosid are not effective in preventing pregnancies like regular ECs on the market such as Levonorgestrel-only pills [50].

A study from Nigeria also reported the likes of Andrews liver salt, thiazamide (MNB 760), Alabukun (local aspirin), salt and water, alcohol, lime, potash, and yoyo bitters being used as EC [38]. This phenomenon of using non-conventional method as EC methods could be as a result of traditionally or locally accepted methods of emergency contraception in SSA. The variety of options used seems to vary from region to region in SSA. This can also be a reason why unintended pregnancy is high in SSA since some of the non-EC methods used cannot prevent pregnancy. A qualitative study conducted in DR Congo also revealed that the participants used other methods and mechanisms as their abridged version of EC. The study reported methods like douching, drinking salted water or sodas, using herbal concoction, jumping hard, antibiotics, deworming medicines (Décaris, Tanzol), and antimalarial medicines (quinine, tetracycline) as EC [39].

The types of EC used were not known by some of the participants and some could not remember the type used. This can also serve as a reason for the low use of EC in SSA. The type of EC used by most of the participants in this review was Postinor 2 [14, 20, 21, 24, 37, 38]. Others included ECPs, and IUCD [16, 17, 21, 24, 25, 28, 29, 35, 40].

These included review articles depict that women of reproductive age might have little knowledge on types of EC available in their region.

### The emergency contraceptive awareness and use dichotomy

This study observed that women in their reproductive age were aware about EC (about 93.5%). Although they generally had high EC awareness levels, some of the included research articles suggested that their awareness lacks depth and this could be attributed to their backgrounds. Among women who have heard of EC were those who have ever used contraceptives and have higher educational qualifications. On the other hand, this was suggesting that people with lower educational background and non-contraceptive users were less likely to use EC method. Other factors that could be associated with level of EC awareness and its use were the place of residence, age of respondents, marital status and nulliparous women. In the case of place of residence, those who reside in urban areas are more likely to know about EC than their counterparts who reside in rural areas and also more likely to have availability of the method to also promote it use. These above factors listed above could be the primary reasons to why the level of EC awareness in SSA were high compared to the level of EC use.

Further studies can be done to find out the effect of demographics such as age, place or residence, type of job and marital status on the Awareness and use of EC to answer questions such as; does the age and income level of a woman in reproductive age affect the use of EC and its awareness, are single women more likely to use EC compared to married women, and factors that influence the uptake of EC in SSA.

Some other studies have examined both awareness and use of EC and identified a gap between awareness and use of EC, for example Jackson et al. (2000) [51] in the U.S.A., Sorensen, Pedersen & Nyrnberg (2000) [52] in Denmark, Aneblom, Larsson, Odlind & Tyden (2002) [53] in Sweden and Korea by Kang & Moneyham (2008) [54]. Such observations (gap between awareness and use of EC) have also been made in Africa such as Nigeria by Zeleke, Zebenay & Weldegerina (2009) [55]; Kagashie et al. (2013) [56], Kolawole, Abubakar & Zaggi (2015) [57], Tanzania by Kagashe et al. [56]. Ghana by OseiTutu, Aryeh-Adjei & Ampadu (2018) [58] and in South Africa by Roberts et al. (2004). In all these studies, a greater proportion of the respondents knew of EC than the proportion that used it. Furthermore, a smaller proportion knew of the recommended time frame for its use. These findings are similar to what this current study is pointing out; EC Awareness is generally higher than usage of EC. The 20–24 years age group generally are more aware of ECs [59–65].

The implication of these the included research articles could be that the respondents used in these studies could be fairly young and mostly unmarried. They are also the ones who are likely to experience unplanned pregnancies and abortions because they have the tendency to be sexually active. Understanding the discrepancy between high awareness levels and underuse of ECs is a subject of public health relevance. Being aware of EC does not guarantee its use. The reasons could be attributed to general lack of in-depth knowledge and misconceptions about their usage. There is also a probability where the context (SSA) in which this method will be used generally frowns upon sexual activities among the youths of the society and hence, women therefore do not want to use the method because it speaks to the society about, they being sexually promiscuous. Further studies could be done to find out the effect of culture, religion and believes on the use of contraceptive methods. Also, studies to understand commonalities and differences between regular contraceptives and ECs, why regular contraceptives are used as ECs in SSA and understanding traditional emergency contraceptives and its effectiveness to prevent unwanted pregnancies.

The identification of non-EC methods used by some of the study participants could also be as a result of some factors such as peer pressure, availability of EC method, cost, and lack of knowledge on EC methods. Further research can be done to find out the factors that contributes or enable the use of non-EC methods in SSA. A comparative study can also be conducted to find out why, certain groups of people prefer and use EC methods and why others do not.

Self-report of EC awareness and use was used in the included review studies. Overall, the highest and lowest rates of both EC awareness EC use were recorded from Ethiopia and Nigeria [29, 36, 40]. This could be explained by the various demographics of the study area participants and place the study was conducted. The study that reported the highest and lowest level of EC awareness and use had participants with demographics such as age, religion, place of stay, number of children, living with, parents’ education, marital status, occupation and educational status. When these studies [29, 36, 40] are compared, it could be suggested that, the rates of EC awareness and use may have been influenced by the demographics of the study population. Each of these demographic variables could cause the rates to increase or decrease. Also, the data collection tools used in the individual included research articles could also have accounted for the rates of EC awareness and use. For example, the study conducted by Tesfaye, Tilahun, and Girma (2012) [29] which recorded both the lowest rates of EC awareness and use, data was collected by researcher administered questionnaires. It could be that the participants were not comfortable sharing their sexual history with a stranger, or feared others might hear of what they are saying. Hence, participants gave answers that might be socially accepted and hence not entirely the true representation of rates of EC awareness and it use. In the context of SSA, where culture and society does not widely accept sexual association to the young and upcoming ones, this could have led to the participants of the studies giving socially accepted responses.

This review has some limitations:

The selected studies used in this study were conducted among different population or similar but at different times and intervals. There is need to adopt a standardized data collection tool to capture awareness and usage patterns of ECs. The inclusion of exclusively articles published in the English language could be a source of bias, as studies published in French, Spanish, Arabic, Portuguese speaking countries were excluded.

## Conclusion

Although variations do exist between countries in SSA according to the year of study, the general level of EC awareness has been increasing. The level of awareness and use of ECs in SSA amongst women of reproductive age (15–49 years) was highest amongst the 20–24 years age group compared to the adolescents (15–19 years) and over 24 years group. However, the level of EC use was lower compared to the level of awareness. Sub - optimal use of ECs is a lost opportunity to prevent unintended pregnancy and unsafe abortions. It is relevant to understand the similarities and differences regarding the facilitators and barriers to the use of ECs and other types of contraceptives to allow for the design of more targeted interventions to improve use. Understanding the mismatch between high levels of awareness and low levels of use of ECs is an important area for future research.

### Recommendations

Given the extremely high rates of awareness of emergency contraceptives in many of the countries studied, programmes should continue to focus on disseminating accurate information about the method, both in the general population and the vulnerable groups and those identified in this review as being unlikely to have heard of it. This will help to dispel any fears of the side effects and other misconceptions about EC and promote its use. Health promotion strategies should also be directed towards improving EC and other contraceptives utilization among the sexually active youth as part of the package of comprehensive reproductive health in tertiary schools. These health promotion campaigns should also make room for people in this age group who are out of tertiary schools. This could improve the awareness level of women of reproductive age (15–49 years) who are still in or out of schools. Promotion and advance provision of EC to the students would very likely enhance their use, just as in developed countries.

It is also recommended that governments, donors, and the non-governmental sectors should focus on meeting the need for ECs to meet the reproductive health needs of women of reproductive age in SSA countries by providing this group of people with ECs for free or at very reduced rates to encourage its usage. Health promotion strategies should be used to sensitize the general population about the need for EC to help reduce medication-related discrimination and stigmatization Further studies should be conducted to help provide more detailed investigations of social, cultural, and economic factors at work in these countries to fully make sense of differences, particularly by age and marital status that influence the use of EC in SSA. Case studies might also be useful in describing how countries such as Ethiopia and Nigeria achieved usage above 30% that was identified in this review.

## Data Availability

All articles included in the final review have been carefully cited.

## Abbreviations

IUCD: Intrauterine contraceptive device
SSA: Sub – Saharan Africa
EC: Emergency contraception
